# Seroprevalence and associated risk factors for chlamydiosis, coxiellosis and brucellosis in sheep and goats in Borana pastoral area, southern Ethiopia

**DOI:** 10.1186/s12917-020-02360-0

**Published:** 2020-05-20

**Authors:** Asamenew Tesfaye, Mesfin Sahele, Teshale Sori, Chala Guyassa, Abebe Garoma

**Affiliations:** 1 National Anima Health Diagnostic and Investigation Center (NAHDIC), P. O. Box 04, Sebeta, Ethiopia; 2 Collage of Agriculture and Veterinary Medicine, Addis Ababa University, P. O. Box, 34 Bishoftu, Ethiopia

**Keywords:** Borana, *C. abortus*, *C. Burnettii*, *Brucella*, Abortion, Sheep, Goats, Risk factors

## Abstract

**Background:**

Abortion is considered an important disease problem of small ruminants in Borana pastoral area. A cross-sectional study was conducted to estimate the prevalence and risk factors of chlamydiosis, coxiellosis (Q-fever) and brucellosis in small ruminants in selected districts of Borana zone.

**Results:**

A total of 506 sheep and goats were tested using serological tests. Fifty (9.88%; 95% CI: 7.42, 12.82), 144 (28.46%; 95% CI: 24.56, 32.61) and none (0.00%; 95% CI: 0.00, 0.59) of them were positive for chlamydiosis, coxiellosis and brucellosis, respectively. History of abortion was recorded in 136 (32.00%; CI: 27.59, 36.67) of sheep and goats in the study area. The logistic regression analysis, however, showed that statistically significant difference ccurred among districts and between the species of small ruminants. The prevalence odd of antibodies against *C. abortus* was significantly lower in Miyo, Dire and Teltelle districts compared to Dillo. The odd of infection with this bacterium was lower in sheep than goats. Similarly the odd of infection with *C. burnettii* was significantly higher in Dillo district than the rest of the districts studied, higher in goats than sheep and higher in adult animals than young ones.

**Conclusion:**

High prevalence of abortion is observed in sheep and goats in the study area. High seropositivity of *C. burnetii* and *C. abortus* in both sheep and goats tested implies risks of human infection by both diseases. Thus, attention needs to be paid to further study of both diseases in animals and humans in the area.

## Background

Abortion is one of the reproductive wastages causing considerable economic losses in small ruminants through loss of fetus and reduced milk production. Abortion in small ruminants is caused by various infectious agents [1, 2], the common ones being *Chlamydophila abortus* (*C. abortus*) [3, 4]*, Coxiella burnetii* (*C. burnetii*) [5] and *Brucella melitensis* [6]. In addition to causing economic losses these diseases also cause human illnesses [7–9].

Chlamydiosis (also known as enzootic abortion) has been recognized since 1950 and is considered worldwide in distribution [10, 11]. Contaminated environment, water and feed are incriminated as source of infection for susceptible animals [12] although aerosol [13] and venereal [11] transmissions are possible. It has been documented that it causes abortion in 25 to 60% of naive sheep and goats in UK and North America. Abortion occurs in primiparous ewes and does during epizootics [14]. The prevalence of abortion was shown to be low in the first year and tend to increase during consecutive years as reported in countries such as Greece and Iran [15]. Epidemiological investigations showed that chlamydiosis occurs in 44% of diagnosed abortion cases in UK, in 56% of the cases in Spain [16] and 69% in Egypt [17].

Coxiellosis (also called Q-fever) is also one of the causes of abortion in small ruminants [18]. An infection with *C. burnetii* is usually latent in livestock though abortion storm as high as 60% has been documented in goats in Netherlands. The prevalence of abortion was 5% in sheep farms in the country [19]. Although it needs to be proved, ticks are incriminated for transmission of Q-fever to susceptible hosts from wild and domestic animals reservoirs [19, 20]. However, the transmission of Q-fever from livestock to humans is similar to that of brucellosis, including the consumption of unpasteurized dairy products, contact with aborted fetuses and other animal products (most often through respiratory and conjunctival routes). Antibodies to Q-fever were found in 54.2% of sera collected in Southeast Ethiopian pastoral goats [21].

Small ruminant brucellosis is a disease of economic and public health impact. It is mainly caused by *Brucella melitensis* (*B. melitensis*) both in sheep and goats [22, 23]. There are three biovars of *B. meltensis* that have differing geographic distribution, but no difference in pathogenicity or animal species affected [19]. Brucellosis in small ruminants is characterized by reproductive wastages such as abortion, stillbirth, birth of weak offspring and infertility [24]. Very low prevalence was reported in different parts of Ethiopia.

Although serological evidence of brucellosis and Q-fever was reported in sheep and goats in Ethiopia, to our knowledge no study has been conducted on chlamydiosis so far. Sheep and goat production is very important activity in Borana pastoral area, where there has been empirical evidence of frequent occurrence of unconfirmed cases of abortion and stillbirth. It is difficult to obtain the magnitude of abortion in the pastoral areas due to lack of records. However, field veterinarians and livestock owners claim frequent occurrence of abortion in sheep and goats. Personal observations during the filed survey also confirmed this. Screening of animals for diseases causing abortion is important to design control methods. Since there is close contact with humans, the results of investigations also has important implications to human health. Therefore, the objective of this study was to screen sheep and goats in selected districts of Borana pastoral zone for Q-fever, chlamydiosis and brucellosis.

## Results

Out the total of 506 sheep and goats tested 50 (9.88%; 95% CI: 7.42, 12.82), 144 (28.46%; 95% CI: 24.56, 32.61) and none (0.00%; 95% CI: 0.00, 0.59) of them were positive for chlamydiosis, coxiellosis and brucellosis, respectively. The proportion of animals testing positive for both chlamydiosis and coxiellosis was higher in adults. The difference between the age groups of animals was statistically significant for *Coxiella* infection although it was only marginally significant for *Chlamydia* infection. For both diseases the odd of infection was higher in Dire, Miyo and Teltelle districts than Dillo (Tables 1 and 2). The prevalence odd of antibodies against *C. burnettii* was significantly lower in Dire (half of Dillo), Miyo (nearly six times less than that of Dillo) and Teltelle (nearly one-third of Dillo). The odd of infection with this bacterium was 0.269 in sheep, which is nearly one-quarter of the odds of infection in goats. Similarly the odd of infection with *C. abortus* was significantly lower in Dire district (OR = 0.439, which is half of Dillo), in Miyo district (OR = 0 .267, nearly one-quarter of Dillo) and Teltelle district (OR = 0 .045, which is 1/22 the odds of infection in Dillo district). The seroprevalence was higher in goats (OR = 2.2 times that of sheep) and adult animals than young ones. However, there was no statistically significant difference between the two genders of the animals in terms of frequency of infection with both diseases.

**Table 1:** The results of sero-prevalence of *C. abortus* infection in sheep and goats in Borana pastoral zone

Variables	N^**0**^ tested	Pos.	Percent	OR	95%	CI	***P***-value
**District**							
Dillo	170	32	18.82				
Dire	113	10	8.85	0.439	0.197	0.979	**0.044**
Miyo	130	7	5.38	0.267	0.107	0.666	**0.005**
Teltelle	93	1	1.08	0.045	0.006	0.337	**0.003**
**Species**							
Goat	293	33	11.26				
Sheep	213	17	7.98	0.469	0.242	0.912	**0.026**
**Age**							
Adult	359	44	12.26				
Young	147	6	4.08	0.417	0.160	1.087	0.074
**Sex**							
Female	424	41	9.67				
Male	82	9	10.98	1.305	0.570	2.987	0.528
Constant				0. 345	0. 207	0. 575	0.000
**Total**	**506**	**50**	**9.88**

**Table 2:** The results of seroprevalence of *C. burnettii* infection in sheep and goats in Borana pastoral zone

Variables	N^**0**^ tested	Pos.	Percent	OR	95%	CI	P-value
**District**							
Dillo	170	72	42.35				
Dire	113	33	29.20	0.508	0.288	0.898	**0.020**
Miyo	130	17	13.08	0.175	0.090	0.338	**0.000**
Teltelle	93	21	22.58	0.317	0170	0.591	**0.000**
**Species**							
Goat	293	104	35.49				
Sheep	213	39	18.31	0.268	0.166	0.433	**0.000**
**Age**							
Adult	359	123	34.26				
Young	147	20	13.61	0.417	0.232	0.750	**0.003**
**Sex**							
Female	424	122	28.77				
Male	82	21	25.61	1.158	0.625	2.148	0.641
Constant				1.575	1.036	2.393	0.033
**Total**	**506**	**144**	**28.46**				

Overall history of abortion was recorded in 136 (32.00%; CI: 27.59, 36.67) of ewes and does in the study area. It was observed in 85 goats (29.01%; CI: 23.88, 34.57%) and 51 sheep (23.94%; CI: 18.38, 30.25%). Of these female animals with history of abortion 12 (8.82%; CI: 4.64, 14.91) of them were positive for anti-*C. abortus* antibodies. In contrast anti- *C. abortus* was detected in 38 of 370 (10.27%; CI: 7.37, 13.82) females with no history of abortion. The occurrence of abortion was not statistically associated with serprevalence of *C. abortus* (OR = 0 .69; CI: 0.34, 1.39). Animals having two or more parities were more prone to be seropositive than nulliparous animals although the difference was not statistically significant. Similarly out of a total of 136 females with history of abortion 38 (27.94%; CI: 20.59, 36.28) of them tested positive for anti-*C. burnettii* antibodies whereas 99 of the 370 (26.76%; CI: 22.31, 31.58) of females with no history of abortion gave positive results for anti-*C. burnettii* antibodies. The odds of seropositivity was 1.14 (CI: 0 .72, 0 1.79) higher in animals with history of abortion than those with no history of abortion. Seropositivity to *C. burnettii* was higher in animals having two or more parties (OR = 2.18; CI: 1.01, 4.75) than nulliparous ones.

## Discussion

This study was conducted in Borana pastoral area where small ruminants provide multitudes of financial, social and collateral services to the community. Hence, the 32% abortion observed has significant impact the livelihood of the pastoral community and the national economy. That is, abortions reduce replacement lambs and kids and affect the off take of young stock in light of the expanding abattoirs. Unless the knowledge, aptitude and practices of the pastoralists is improved, it can significantly affect the small ruminant industry. Optimum control of diseases causing abortion requires the understanding of their etiology and epidemiology. In this regard, we screened small ruminants for chlamydiosis, coxiellosis and brucellosis from selected districts of Borana pastoral area where abortion has been reported frequently. The study provided evidence of occurrence of chlamydiosis and coxiellosis in the area.

To the best of our knowledge this the first report of occurrence of infection with *C. abortus* in small ruminants in Ethiopia. The prevalence of anti- *C. abortus* antibody observed in sheep and goats in this study is in agreement with the report of Ghorbanpoor and colleague [25] who observed prevalence of 9% in sheep. However, the prevalence reported in this study is lower those reported elsewhere in the world such as that of José and colleague [26] from Brazil, Qudah and colleague [27] from Jordan, Esmaeili and colleague [28] from Iran and Vidal et al. [29] from Switzerland. Our result is also lower than the reports documented in in The Netherlands where 17% of abortion cases in goats were associated with *Chlamydia* species [30]. The difference observed in prevalence of chlamydiosis could be due to difference in the production system and the laboratory method used.

The results of logistic regression analysis showed that the seroprevalence of chlamydiosis is higher in goats than sheep. This could due to genetic variation in the predisposition to *C. abortus* between sheep and goats [31]. Statistically significant difference was also observed among districts. This might be ascribed to difference in local husbandry practices and frequency of contact with various flocks and herds as districts such as Dire, Teltelle and Miyo border Kenya, in which livestock movement in search of pasture and water is frequent. This movement can introduce and spread diseases. In addition there is a big livestock market in Dire different animals species are marketed. In this market place small ruminants from different districts in Borana zone and some neighboring districts of Kenya gather together weekly. This might have favored spread of the bacteria among animals, which made the prevalence to be higher. In contrast, Teltele, where the lowest prevalence was observed is found further from the market center. This is in agreement with the previous reports of Qudah et al. [27] who also observed significant difference in the seroprevalence of *C. abortus* infection among different locations.

The first evidence of occurrence of infection of Ethiopian livestock with *C. burnetii* dated back to the reports of Philip et al. [32] who observed seropositive cattle, sheep and goats. Later investigation carried out in pastoral livestock population revealed seroprevalence of 54.20% in goats [21]. This is higher than the prevalence reported in this study. The difference observed could be due to difference in study animals used. The previous authors used randomly selected animals that were seropositive to brucellosis, whereas we sampled animals from the general small ruminant population. However, studies conducted elsewhere in Africa and in the world are in agreement with our observation. For instance seroprevalence in the range of 23 to 33%, 15 to 33% and 24% has been documented in Egypt, Morocco and Sudan, respectively [31].

Similarly seroprevalence ranging from 24.66 to 31.97% [33] and 29.80 to 36.50% [34] has been reported in small ruminants from Iran. Vandenburg and colleague [31], however, reported lower prevalence ranging from 7 to 12% in Tunisia and 11% in Ghana. That is, infection with *C. burnetii* (Q fever) is widespread in the world with varying prevalence. The variation in prevalence among countries highlights the importance of understanding risk factors which may operate at a local scale. The seroprevalence of infection with *C. burnetii* varies significantly among districts for similar reasons described above. In consent to our observation similar difference in the prevalence of Q fever has been reported among localities by Nakoune et al. [35] from Central African Republic. Significant difference has also been reported between adult and young animals and between species of animals [21]. The higher prevalence in goats than sheep could be due to genetic susceptibility or host preference of tick vectors although this observation needs to be further elucidated. The higher prevalence in adults can be ascribed to cumulative effects of age in which older animals became exposed to the vectors and acquire *C. burnetii* infection to which they develop antibodies.

The low seroprevalence of brucellosis observed in this study is remarkable considering the high prevalence reported previously in pastoral herds of goats in south eastern Ethiopia. Since only 506 animals from three districts were sampled positive animals might have been missed in the prevalence in the herds or flocks is low. On the other hand it is becoming a general trend for bovine brucellosis to be very low in Ethiopia [36].

## Conclusion

High prevalence of abortion is observed in sheep and goats in the study area. High seropositivity of *C. burnetii* and *C. abortus* in both sheep and goats tested implies risks of human infection by both diseases. Thus, attention needs to be paid to further study of both diseases in animals and humans in the area. Since our study is a cross-sectional serological survey, the observed abortion in seropositive animals does not imply that *C. burnetii* and *C. abortus* have caused the abortion. This needs to be elucidated from longitudinal studies.

## Methods

### Study areas

**Fig. 1:**
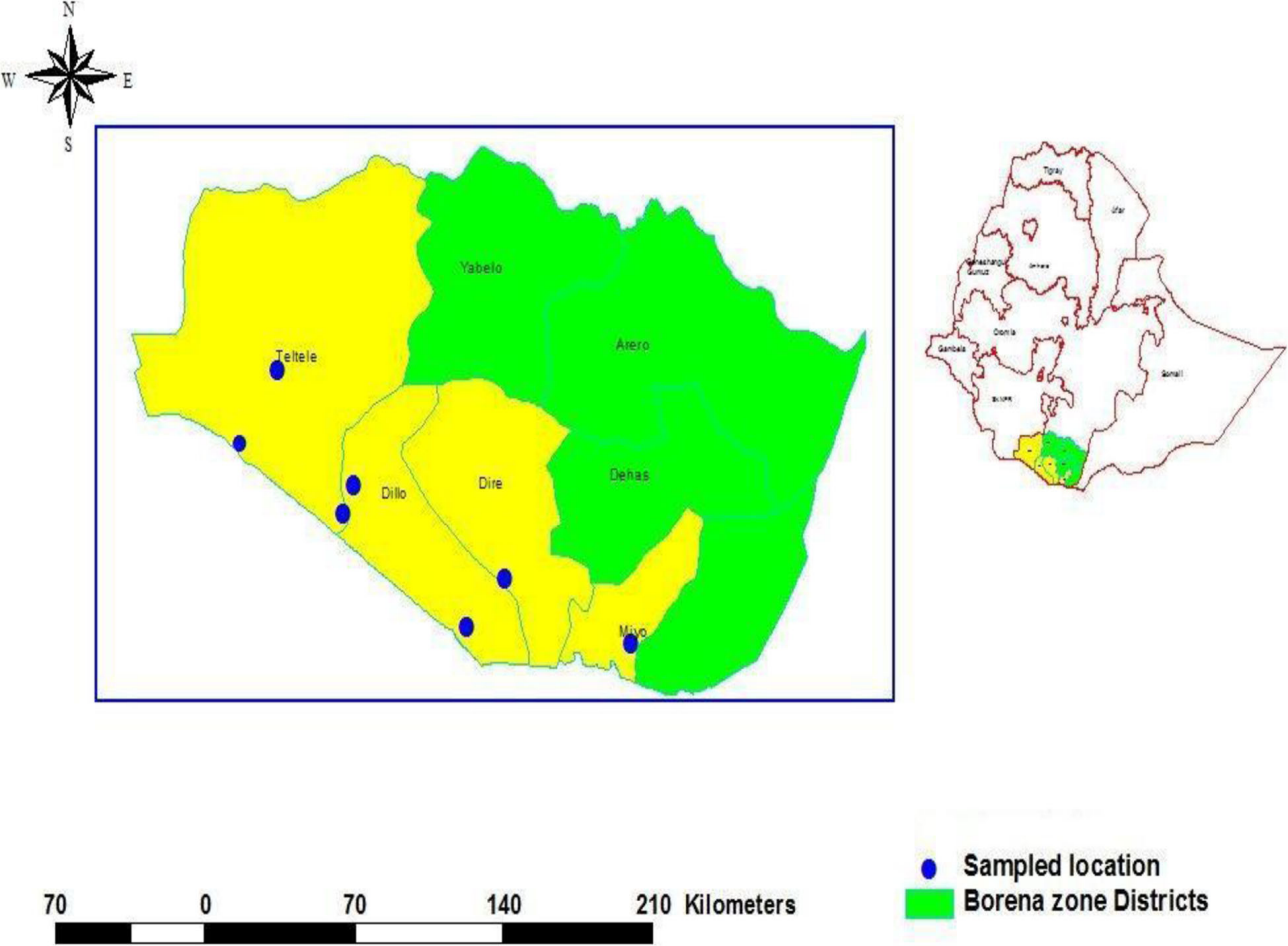
Map of Ethiopia and Oromia Regional State depicting the location of study area (Source: the map was obtained from Borana Zone Administration with permission)

The study was conducted in Borana pastoral districts in Borana zone, southern Oromia, Ethiopia. Borana zone is located between 3°36′ – 6°38′ North latitude and 3°43′- 39°30′ East longitude in southern Ethiopia bordering Kenya. The zone is divided into 13 districts covering a total area of 48,360 km. Borana Zone has a total population of 962,489 in a total of 182,258 households making an average of 5.28 persons to a household. The zone is characterized by a semiarid to arid climate. The altitude of Borana zone ranges from 1000 to 1700 m above sea level featured by isolated mountains and valleys. The mean annual rainfall of the area ranges from 250 to 700 mm. The annual mean temperature varies from 19 to over 25 °C. Extensive pastoralism (nomadic pastoralism) is the main means of livelihood for the Borana people [37]. Cattle, goats, sheep and camels are important livestock species raised in the area. Four districts namely Dillo, Dire, Mio and Teltele (Fig. 1) were selected purposively for this study. They were selected because there was frequent abortion in both sheep and goats as reported by field veterinarians and also from personal observation during the field survey. The districts are bordered by Kenya and there is a big market in Dire where ruminants are marketed from adjacent districts of Borana and Kenya. That is, there is frequent movement of animals in the area in search of market and pasture.

### Study design and sampling

A cross-sectional study was conducted in sheep and goats from the selected districts. The sample size required was computed using the formula described by Thrusfield [38]. No pre-specified prevalence considered for each pathogen since no previous study has been undertaken in the area. To increase the number animals we assumed the expected prevalence to be 50%. According to the formula a minimum of 384 animals (192 sheep and 192 goats) were needed considering both species as one epidemiological unit. However, since sufficient collection materials and reagents were available the number was increased to 293 goats and 213 sheep making a total of 506 samples. Eighty-two of the study animals were male whereas 424 of them were female. A three-stage sampling method was employed in which the study districts were selected purposively as described above. This was followed by random selection of villages among all villages registered under the selected districts. From each district four villages were selected randomly. From each villages four farms (properties) having more than 10 sheep and goats aged greater than 3 months were selected from four villages. None of the animals in the study area were vaccinated against any of the diseases studied as there are no vaccines used against these diseases in Ethiopia.

### Blood sample collection

Approximately 5–7 mL of blood samples were collected from jugular vein each sheep and goat for serological examination using sterile plain vacutainer tubes and needles following the necessary ethics described under the ethical declaration section. The tubes were labeled individually and were kept in icebox and transported to Yabello Pastoral and Dryland Agriculture Research Center. The samples were allowed to stand overnight to allow serum separation. The sera were separated after centrifugation at 1500 g for 10 min. The sera were then collected into sterile cryogenic tubes and stored at − 20 °C until transportation to National Animal Health Diagnostic and Investigation Center, Sebeta, Ethiopia for analysis. The shipment of the samples was done using an ice box with ice pack. Various variables such as flock size (count), animal species (binary), flock composition, age (binary; as adult and young) and sex of animals (binary), location, number of pregnant animals (count), occurrence of abortion (binary) and stillbirth (binary) were recorded during blood collection by the investigators. Information on some of the variables such as abortion, stillbirth and age of animals were obtained from the owners.

### Laboratory analysis of samples

#### Chlamydiosis

##### Enzyme linked immunosorbent assay (ELISA)

A commercial indirect ELISA was used to detect antibodies against *C. abortus* at National Animal Health Diagnostic and Investigation Center (NAHDIC), Sebeta, Ethiopia having sensitivity and specificity of 96.8 and 100%, respectively [39]. The test was conducted in microplate coated with inactivated antigen following manufacturer’s instruction. One hundred μL of pre-diluted sera and controls (1:400) were added into microtiter plate and incubated for one hour at 37 °C. The plate was then washed 3 times. Then 100 μL of conjugate was added to each well followed by covering of the plates before incubating it for 1 h at 37 °C. The plates were washed 3 times. Finally 100 μL of substrate was added to each well and incubated at 18–26 °C for 15 min. Then 100 μL of stop solution was added to each well and the result was read at a wavelength of 450 nm. The test is valid if the mean Optical density (OD) for negative controls (≤0.500), positive control (≤ 2.00) and the difference between them (≥0.300). Sample to positive ratio (S/P %) was computed as the difference between the sample absorbance and the absorbance of negative control divided by the difference between absorbance of positive and negative controls and multiplied by 100. The result is interpreted as negative if S/P ratio is < 30, suspected if S/P ratio is ≥30 but < 40 and positive if S/P ratio is ≥40. Both positive and negative controls were provided along with the kits and validation was carried out as described by the manufacturers of the kit.

#### Brucellosis

##### Rose Bengal plate test (RBPT)

All sera samples collected were initially screened by RBPT using RBPT antigen (Animal and Plant Health Agency, New Haw, Addlestone, Surrey, KT15 3NB, United Kingdom) according to OIE procedures [5]. Briefly, RBT antigen (30 μL) was added onto a glass slide next to 90 μL of sheep and goat sera. The antigen and test serum were mixed thoroughly in a plastic applicator, shaken for 4 min, and agglutination was read immediately. Any observed agglutination by the naked eye was considered to be a positive reaction.

##### Complement fixation test (CFT)

All samples that were RBPT-positive were further subjected to complement fixation test as aconfirmatory test at the National Veterinary Institute (NVI), Bishoftu, Ethiopia. The Brucella antigen and control sera (positive and negative) used during the test were produced by Veterinary Laboratory Agency, UK. The standardization of the antigen was made at 1:20 working dilution (strength). The Brucella antigen, complement and 3% sensitized sheep red blood cells were added after the test sera were serially diluted (1:5, 1:10, 1:20, and 1:40) in microtitre plates. Then the plates were incubated at 37 °C for 30 min. The test was considered positive when the reading was as partial fixation (50% haemolysis) or complete fixation (no haemolysis) at 1:10 dilution. The validity of the test was considered when there was complete hemolysis in negative control serum and the positive control shows inhibition of hemolysis.

Although the sensitivity and specificity of both tests are lacking in tropical settings, the values for these parameters were considered from reports made elsewhere in the world. Hence, for RBPT: sensitivity ranges from 91 to 100% in affected areas [40], and from 96.7 to 100% on *Brucella*-free farms [41]; specificity from 95 to 99% in affected areas [40], and from 79 to 91.9% on *Brucella*-free farms [41] – for the CFT: sensitivity from 96.7 to 100% and specificity from 88.8 to 97.7% [41].

#### Coxiellosis

For detection of antibodies against *Coxiella burnettii* in sera samples a commercial enzyme-linked immunosorbent assay was used following the manufacturer’s instruction. The test has a very high sensitivity and specificity as reported by the manufacturer [42]. Briefly sera samples, negative and positive controls were diluted at 1/400 using wash solution. The control samples (provided along with the kits) were dispended into duplicate wells whereas the sera samples were added to the remaining wells and the plate covered and shaken gently before it was incubated for 60 minutesat 37 °C in humid chamber. The wells were emptied and washed three times with 300 μL of wash solution and 100 μL of conjugate was added and incubated as described above. The content of the plate was discarded and washed three times with 300 μL of wash solution and 100 μL of substrate was added. The plate was incubated at room temperature for 15 min and 100 μL of stop solution and the result was read at 450 nm wavelengths. Validation of the test, computation of sample to positive ration and final interpretation of the result was performed as described above.

### Statistical analysis

The effects of various variables such as district, species, age (categorized as young and adult), sex and parity on seropositivity to *C. abortus* and *C. burnetii* was analyzed by multivariable logistic regression analysis using STATA Version 13 (StataCorp, 4905 Lakeway Drive, College Station, Texas 77,845 USA). Since our outcome variables were binary the logit link was used to model the association between the occurrence diseases as a function of various predictors. The logistic display function was used to compute odds ratio from the model coefficients to estimate the effect size. The parameters describing relationship between predictors and sero-prevalence was estimated using maximum likelihood estimation method. Mixed-effects logistic regression was used to assess if clustering effect has occurred due to district and farms but there was no clustering effect. The effects of parity and abortion on sero-prevalence were analyzed only for female animals. In all cases differences observed were considered statistically significant when *P* < 0.05.

## Data Availability

All data generated and analyzed during this study are included in Tables 1 and 2. However, the raw data is available from the corresponding author upon reasonable request.

## Abbreviations

CFT: Complement Fixation Test
CI: Confidence Interval
ELISA: Enzyme Linked Immunosorben Assay
NAHDIC: National Animal Health Diagnostic and Investigation Center
NVI: National Veterinary Institute
OD: Optical Density
OIE: World Organization for Animal Health
OR: Odds Ratio
RBPT: Rose Bengal Plate Test
S/P: Sample to Positive Ratio
